# Skeletal muscle proteome analysis underpins multifaceted mitochondrial dysfunction in Friedreich’s ataxia

**DOI:** 10.3389/fnins.2023.1289027

**Published:** 2023-10-31

**Authors:** Elisabetta Indelicato, Klaus Faserl, Matthias Amprosi, Wolfgang Nachbauer, Rainer Schneider, Julia Wanschitz, Bettina Sarg, Sylvia Boesch

**Affiliations:** ^1^Center for Rare Movement Disorders Innsbruck, Department of Neurology, Medical University of Innsbruck, Innsbruck, Austria; ^2^Institute of Medical Biochemistry, Protein Core Facility, Medical University of Innsbruck, Innsbruck, Austria; ^3^Institute of Biochemistry, Center of Molecular Biosciences Innsbruck (CMBI), Leopold-Franzens University Innsbruck, Innsbruck, Austria; ^4^Laboratory of Tissue Diagnostics, Department of Neurology, Medical University of Innsbruck, Innsbruck, Austria

**Keywords:** Friedreich’s ataxia, skeletal muscle, mass spectrometry, proteomics, mitochondria, frataxin

## Abstract

Friedreich’s ataxia (FRDA) is a severe multisystemic disorder caused by a deficiency of the mitochondrial protein frataxin. While some aspects of FRDA pathology are developmental, the causes underlying the steady progression are unclear. The inaccessibility of key affected tissues to sampling is a main hurdle. Skeletal muscle displays a disease phenotype and may be sampled *in vivo* to address open questions on FRDA pathophysiology. Thus, we performed a quantitative mass spectrometry-based proteomics analysis in gastrocnemius skeletal muscle biopsies from genetically confirmed FRDA patients (*n* = 5) and controls. Obtained data files were processed using Proteome Discoverer and searched by Sequest HT engine against a UniProt human reference proteome database. Comparing skeletal muscle proteomics profiles between FRDA and controls, we identified 228 significant differentially expressed (DE) proteins, of which 227 were downregulated in FRDA. Principal component analysis showed a clear separation between FRDA and control samples. Interactome analysis revealed clustering of DE proteins in oxidative phosphorylation, ribosomal elements, mitochondrial architecture control, and fission/fusion pathways. DE findings in the muscle-specific proteomics suggested a shift toward fast-twitching glycolytic fibers. Notably, most DE proteins (169/228, 74%) are target of the transcription factor nuclear factor-erythroid 2. Our data corroborate a mitochondrial biosignature of FRDA, which extends beyond a mere oxidative phosphorylation failure. Skeletal muscle proteomics highlighted a derangement of mitochondrial architecture and maintenance pathways and a likely adaptive metabolic shift of contractile proteins. The present findings are relevant for the design of future therapeutic strategies and highlight the value of skeletal muscle-omics as disease state readout in FRDA.

## Introduction

Friedreich’s ataxia (FRDA, OMIM #229300) is a rare multisystemic disorder with a prevalence of up to 1:25,000 in the Caucasians (Vankan, 2013). The disease is triggered by a deficiency of the nuclear-encoded, mitochondrial protein Frataxin (FXN) (Campuzano et al., 1996). The majority of patients carry biallelic GAA-repeat expansions within the first intron of *FXN* gene (Campuzano et al., 1996), an alteration which represses the transcription at this locus but does not affect protein structure (Gottesfeld, 2019). Frataxin is involved in the biosynthesis of iron-sulphur clusters (ISC), the prosthetic groups of several respiratory chain subunits and DNA repair enzymes (Rotig et al., 1997; Chen et al., 2002; Shan et al., 2007; Thierbach et al., 2010). Established consequences of frataxin deficiency are defective ISC synthesis, impaired oxidative phosphorylation (OXPHOS) and mitochondrial failure with iron accumulation (Gonz Alez-Cabo and Palau, 2013). Despite its ubiquitous expression, frataxin deficiency results in the clinical involvement of few organs (Parkinson et al., 2013). A progressive, mainly afferent, ataxia is the universal clinical feature, which reflects the involvement of dorsal root ganglia, posterior column of the spinal cord, peripheral sensory nerves, and deep cerebellar nuclei (Parkinson et al., 2013). Up to 75% of patients are affected by cardiomyopathy (Parkinson et al., 2013). Diabetes mellitus is considered a further typical manifestation and occurs in up to 8.7% of patients (Tamaroff et al., 2022). Several aspects of FRDA pathophysiology remain poorly understood. Why few organs are susceptible to frataxin deficiency and which factors eventually drive clinical progression are still open questions.

Functional and histological studies demonstrate that FRDA also features a skeletal muscle involvement (Lodi et al., 1999; Vorgerd et al., 2000; Sival et al., 2011; Nachbauer et al., 2012). Myopathy in FRDA likely contributes to the marked fatigue, exercise intolerance and weakness (Sival et al., 2011). Differently from the key affected nervous tissues, skeletal muscle is accessible to sampling *in vivo*. Bearing this in mind, we previously performed RNA-sequencing in skeletal muscle biopsies obtained from genetically confirmed FRDA patients to gain further insight in the disease pathogenesis. Skeletal muscle transcriptomics recapitulated key pathophysiological aspects of FRDA, such as profound mitochondrial failure and a shift toward transcriptional repression (Indelicato et al., 2023). Changes of mitochondrial transcriptome in skeletal muscle were more extensive than in FRDA patients derived blood cells, fibroblasts or induced pluripotent stem cells (iPSC) derived sensory neurons (Coppola et al., 2011; Napierala et al., 2017; Lai et al., 2019). We furthermore showed that skeletal muscle transcriptomics was sensitive to change induced by pharmacological intervention (Indelicato et al., 2023), pointing to its potential as real-time biomarker for early phase trials.

Beyond the transcriptional control, multiple, additional layers of regulation shape the final protein assortment of a tissue at a given time point (Liu et al., 2016). To generate a complete picture, we thus extended our previous study by performing a mass spectrometry-based proteomics analysis applying isobaric labeling via tandem mass tag (TMT) reagents in skeletal muscle biopsies from FRDA patients and controls. Skeletal muscle proteome analysis in FRDA substantiates a leading mitochondrial signature, which expands beyond the impairment of OXPHOS to involve mitochondrial architecture control and maintenance pathways. Such changes are accompanied by a likely adaptative shift of the muscle specific proteomics, previously not evident at mRNA level (Indelicato et al., 2023). Our findings corroborate a role for skeletal muscle biopsies as valuable surrogate for culprit pathways and biomarkers discovery in FRDA.

## Methods

### Study population and ethical issues

Genetically confirmed FRDA patients were recruited within a registered clinical trial at the Center for Rare Movement Disorders of the Medical University of Innsbruck. Within the trial, biopsies from the gastrocnemius muscle were obtained before and after treatment with rhuEPO. For the present study, we analyzed baseline skeletal muscle biopsies, sampled before rhuEPO treatment. Study protocol including detailed processing of the biopsies has been published elsewhere (Nachbauer et al., 2012). Skeletal muscle biopsies with unremarkable findings from age-matched controls were available for comparison. Patients and controls gave written informed consent to the study and for publication. The study was approved by the local ethic committee (Approval number, UN 3152_LEK). Histology, immunohistochemistry, as well as transcriptomics findings from the biopsies has been previously published (Nachbauer et al., 2012; Indelicato et al., 2023).

### Proteomics procedures

#### Sample preparation

Skeletal muscle biopsies were transferred into 500 μl of ABC-buffer (ammonium-bicarbonate buffer, 100 mM, pH 8.0) containing 0.5% sodium deoxycholate. Proteins were extracted using a Precellys Evolution homogenizer in combination with 2.8 mm metal beads for 3 × 30 s. Protein quantification was performed via Bradford Assay with ROTI^®^-Nanoquant K880 (Carl Roth). Sample volumes equivalent to 50 μg of protein were diluted to 20 μl using ammonium-bicarbonate buffer. Proteins were reduced by adding 20 μl 10 mM dithiothreitol in ABC-buffer followed by incubation at 56°C for 30 min and digested with 1 μg of trypsin (Sequencing Grade Modified Trypsin, P/N: V5111, Promega) overnight at 37°C under agitation. Free cysteines were alkylated by adding 50 μl 550 mM iodoacetamide in ABC-buffer followed by agitation at room temperature for 20 min in the dark. Peptides were buffer exchanged to 100 μl of 100 mM TEAB-buffer (pH 8.5) using Pierce Peptide Desalting Spin Columns (P/N 89852, Thermo Scientific) and TMTpro labeled (TMTpro™ 16plex Label Reagent Set, P/N A44521, Thermo Scientific) according to the manufacturer’s instruction. Fourteen different peptide samples were prepared thus two label reagents (129C and 134N) were not required for this study. All samples were pooled together, lyophilized to dry and redissolved in 60 μl 0.1% formic acid. Peptides were fractionated by high pH reversed-phase chromatography using a XBridge Peptide BEH C18 column, 4.6 mm × 250 mm, 300 Å, 5 μm (P/N 186003625, Waters). Solvents for HPLC were 10 mM formic acid in 2% acetonitrile, pH 10 (solvent A) and 10 mM formic acid in 98% acetonitrile, pH 10 (solvent B). At a constant flow rate of 500 μl/min the following gradient was applied: 0–5% solvent B in 10 min, 5–35% in 60 min, 35–70% in 15 min, held at 70% B for 10 min, increased to 100% in 5 min. A total of 47 fractions were collected. These fractions were recombined into 16 pools (as indicated by the MS raw data file), lyophilized and stored dry at −20°C. Peptides were dissolved in 0.1% formic acid and 5% per pool subjected to nanoscale liquid chromatography coupled to tandem mass spectrometry (nanoLC-MS).

#### Liquid chromatography coupled to tandem mass spectrometry

Pooled peptide fractions were analyzed using an UltiMate 3000 RSCL nano-HPLC system coupled to an Orbitrap Eclipse mass spectrometer (Thermo Scientific) equipped with a Nanospray Flex ionization source as described previously (Kellnerová et al., 2023). In brief, peptides were separated on a homemade column (100 μm i.d. × 17 cm length) packed with 2.4 μm C18 material (Reprosil, Dr. A. Maisch HPLC GmbH). Solvents for nano-HPLC were 0.1% formic acid (solvent A) and 0.1% formic acid in 85% acetonitrile (solvent B). At a flow rate of 300 nl/min the concentration of solvent B was increased from 4 to 30% in 113 min and from 30 to 100% in 5 min. The Orbitrap Eclipse mass spectrometer was operating in the data dependent mode with a cycle time of three seconds. Survey full scan MS spectra were acquired from 400 to 1,600 m/z at a resolution of 120,000 with an isolation window of 0.7 mass-to-charge ratio (m/z), a maximum injection time (IT) of 50 ms, and automatic gain control (AGC) target 400,000. The MS2 spectra were measured in the Orbitrap analyzer at a resolution of 50,000 with a maximum IT of 200 ms, and AGC target or 100,000. Fragmentation was performed by higher-energy collisional dissociation with normalized collision energy of 35.

### Data analysis

The MS data files were processed using Proteome Discoverer version 2.5 (Thermo Scientific). MS/MS spectra were searched by Sequest HT engine against a UniProt human reference proteome database (last modified 25/07/2023). The search parameters were as follows: Enzyme specificity was set to trypsin with two missed cleavages being allowed. Fixed modification was carbamidomethyl on cysteine; variable modifications were oxidation of methionine, phosphorylation of serine, threonine and tyrosine, and acetylation and/or methionine loss of the protein N-terminus. Precursor mass tolerance was set to 10 ppm; fragment mass tolerance was 20 mmu. Maximum false discovery rate (FDR) for protein and peptide identification was set to 1%. For quantitation, the protein fold changes were calculated based on the fourteen TMTpro reporter ion intensities present in MS2 scans. The reporter ion intensities were extracted using the default software settings. Comparison of protein abundances between FRDA and controls was performed by means *t*-test. Differentially expressed (DE) proteins interactions were investigated interrogating the STRING database (Szklarczyk et al., 2015).

### Data sharing

The MS-proteomics data have been deposited to the ProteomeXchange Consortium^1^ via the PRIDE partner repository with the dataset identifier PXD044554.

## Results

**FIGURE 1:**
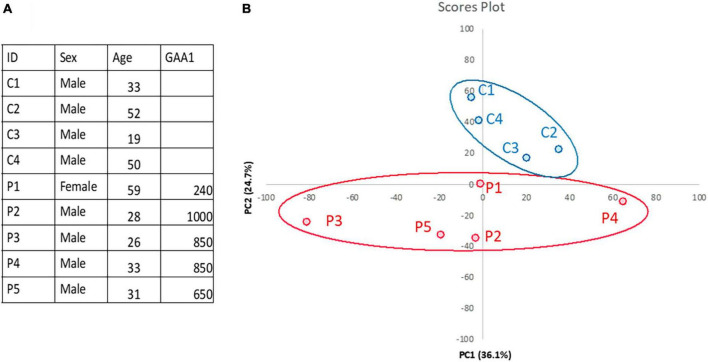
Skeletal muscle proteomics in FRDA and controls. **(A)** Demographics and clinical data of FRDA patients and controls are reported in the table. GAA1, shorter GAA repeat expansion. **(B)** Principal component analysis (PCA) showing clustering of samples from FRDA patients (red) and controls (blue). PCA was performed via ClustVis (https://doi.org/10.1093/nar/gkv468).

Gastrocnemius muscle biopsies from five FRDA patients and four controls were available for the present analysis. Demographic data of patients and controls are reported in Figure 1A. All patients carried two expanded *FXN* alleles. Median disease duration was 15 years (range 7–30). In four out of five patients, disease onset occurred <25 years of age. At the time of the examination, two patients were wheelchair bound and three were ambulatory with walking aid devices. None of them had diabetes mellitus. Two patients displayed mild hypertrophic cardiomyopathy.

**FIGURE 2:**
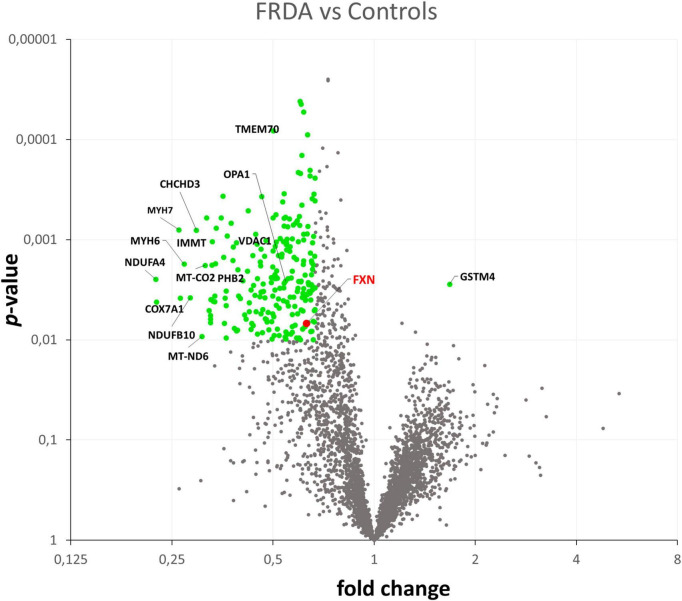
Volcano plot showing in green the DE proteins in the comparison between FRDA and controls.

**FIGURE 3:**
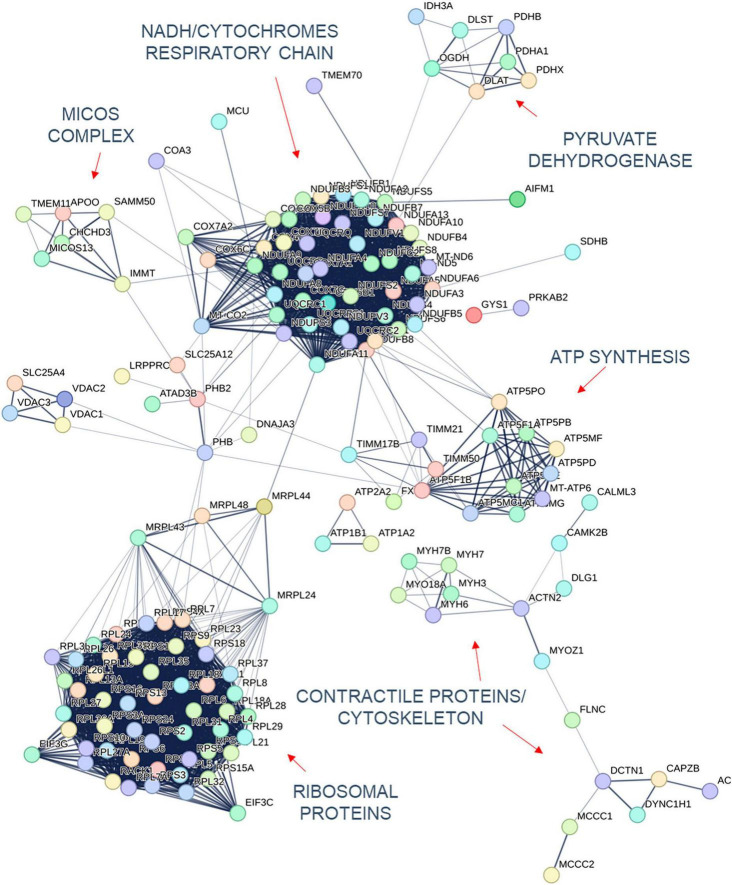
Interactome of the DE proteins in FRDA patients adapted from the STRING database.

Quantification of protein expression levels in FRDA- and control-derived skeletal muscle identified 4,169 protein entries. Considering entries with both *p* < 0.01 and abundance fold change >1.5 in the comparison between FRDA and controls, we identified 228 significant DE proteins (see Figure 2), of which 227 were downregulated in FRDA. Detailed proteomics data of the whole DE set can be found in Supplementary material. Concerning the significant DE proteins explicitly cited in the text, detailed data are reported also in Table 1. Principal component analysis (PCA) showed a clear separation between FRDA and control samples (Figure 1B). Skeletal muscle frataxin displayed a ∼1.5-fold decrease in FRDA as compared to controls (abundance ratio = 0.63, *p* = 0.007). Notably, in our previous transcriptomics study *FXN* mRNA was about ∼3.5-fold decreased in the same skeletal muscle samples comparing to controls (Indelicato et al., 2023). Interactome of the DE proteins based on STRING analysis reflects clustering of DE proteins in several mitochondrial pathways, ribosomal proteins, and muscle specific items (Figure 3). For 169/228 (74%) DE proteins there is evidence of an interaction as target gene of the transcription factor nuclear factor-erythroid 2 (NRF2) (Liska et al., 2022).

**TABLE 1:** Differentially expressed skeletal muscle DE proteins in FRDA as compared to controls.

		Proteomics						
Gene	Description	UniProt accession	Abundance controls	Abundance FRDA	Ratio	Ratio log_2_FC	Statistics	RNA log_2_FC
ACAT1	Acetyl-CoA acetyltransferase 1	P24752	44,667	19,191	0.43	-1.22	0.008	
APOO	Apolipoprotein O	Q9BUR5	3,410	1,209	0.35	-1.50	>0.001	-1.25
ATP2A2	ATPase sarcoplasmic/endoplasmic reticulum Ca2+ transporting 2	P16615	47,706	19,035	0.40	-1.33	0.004	
CHCHD3	Coiled-coil-helix-coiled-coil-helix domain containing 3	Q9NX63	16,272	4,808	0.30	-1.76	0.001	-1.18
CPT1B	Carnitine palmitoyltransferase 1B	Q92523	3,977	1,440	0.36	-1.47	0.003	
FXN	Frataxin	Q16595	3,523	2,215	0.63	-0.67	0.007	-1.86
GSTM4	Glutathione S-transferase mu 4	Q03013	621	1,220	1.68	0.74	0.003	
IMMT	Inner membrane mitochondrial protein	Q16891	54,236	17,850	0.33	-1.60	0.001	-1.05
MICOS13	Mitochondrial contact site and cristae organizing system subunit 13	Q5XKP0	9,199	3,109	0.34	-1.56	0.001	
MT-ND6	Mitochondrially encoded NADH:ubiquinone oxidoreductase core subunit 6	P03923	334	103	0.31	-1.71	0.009	
MYH3	Myosin heavy chain 3	P11055	2,254	734	0.33	-1.62	0.006	2.24
MYH6	Myosin heavy chain 6	P13533	2,154	585	0.27	-1.88	0.002	
MYH7	Myosin heavy chain 7	P12883	818,464	214,590	0.26	-1.93	0.001	
MYH7B	Myosin heavy chain 7B	A7E2Y1	312	105	0.34	-1.58	0.004	
NNT	Nicotinamide nucleotide transhydrogenase	Q13423	56,437	18,560	0.33	-1.60	0.002	
OPA1	OPA1 mitochondrial dynamin like GTPase	O60313	6,923	3,771	0.54	-0.88	0.003	
PDHA1	Pyruvate dehydrogenase E1 subunit alpha 1	P08559	41,959	20,700	0.49	-1.02	0.002	
PDHB	Pyruvate dehydrogenase E1 subunit beta	P11177	29,384	13,574	0.46	-1.11	>0.001	-1.03
PDHX	Pyruvate dehydrogenase complex component X	O00330	16,031	7,552	0.47	-1.09	0.002	
PHB1	Prohibitin 1	P35232	25,071	8,447	0.34	-1.57	0.002	
PHB2	Prohibitin 2	Q99623	30,669	12,453	0.41	-1.30	0.003	1.06
SAMM50	SAMM50 sorting and assembly machinery component	Q9Y512	6,147	3,682	0.60	-0.74	0.001	
SLC25A11	Solute carrier family 25 member 11	Q02978	24,242	9,416	0.39	-1.36	0.008	-0.87
SLC25A12	Solute carrier family 25 member 12	O75746	29,058	16,123	0.55	-0.86	>0.001	-0.77
SLC25A20	Solute carrier family 25 member 20	O43772	2,840	1,837	0.65	-0.62	>0.001	
SLC25A3	Solute carrier family 25 member 3	Q00325	20,637	6,842	0.33	-1.59	0.004	-1.65
SLC25A4	Solute carrier family 25 member 4	P12235	62,067	20,813	0.34	-1.58	0.004	-1.03
TIMM17B	Translocase of inner mitochondrial membrane 17B	O60830	777	307	0.39	-1.34	0.004	
TMEM11	Transmembrane protein 11	P17152	3,517	1,283	0.36	-1.45	0.001	0.98
TMEM70	Transmembrane protein 70	Q9BUB7	909	455	0.5003	-1.42	0.0001	
TNNT1	Troponin T1, slow skeletal type	P13805	50,362	19,275	0.38	-1.39	0.008	
VDAC1	Voltage dependent anion channel 1	P21796	35,183	13,687	0.39	-1.36	0.001	-0.66
VDAC2	Voltage dependent anion channel 2	P45880	18,137	6,892	0.38	-1.40	0.001	-0.85

Differential expression was defined by a *p* < 0.01 and fold change >1.5 in the comparison. Significant changes in the correlating transcript as detected in a previous study (Indelicato et al., 2023) are reported in the last column. *NRF2* targets are highlighted in gray. Log2FC, log_2_ fold change.

### Mitochondrial DE proteins are involved in energy production and structural maintenance

The largest group of mitochondrial DE proteins comprised structural components of all the complexes of the OXPHOS. Notably, downregulated terms referred not only to ISC containing proteins, but also to several structural subunits of complex V/F_o_F_1_ ATP synthase including its assembly factor TMEM70 (see Figure 3). Structural elements of the pyruvate dehydrogenase components (PDHA1, PDHB, and PDHX) were downregulated as well. One distinct mitochondrial DE cluster (CHCHD3, IMMT, MICOS13/C19orf70, TMEM11, and APOO) encompassed proteins functionally involved in the MICOS complex, a large protein apparatus located at the inner mitochondrial membrane which plays a pivotal role in the shaping of cristae morphology. Further interactors and regulators of these processes such as SAMM50 and OPA1 were also downregulated. Notably OPA1 variants can cause inherited optic neuropathy, which occurs isolated or along a broader phenotype with sensory ataxia, sensorineural deafness and myopathy (Hudson et al., 2008), resembling the full-blown phenotype of advanced FRDA. A role in the scaffolding of the mitochondrial tubular network has been unveiled recently also for the DE proteins PHB1/prohibit 1 and PHB2/prohibit 2 (Yoshinaka et al., 2019). A few mitochondrial transporter proteins (SLC25A11, SLC25A4, SLC25A20, SLC25A12, and TIMM17B at the inner membrane and SLC25A3, VDAC1, and VDAC2 located at the outer membrane) listed to the DE downregulated genes. SLC25A11, SLC25A4, and VDAC1 are known to be involved in mitochondrial fission/mitophagy pathways downstream of PINK1/PRKN signaling (Alto et al., 2002; Gallo et al., 2011; Ham et al., 2020).

### Skeletal muscle specific DE proteins reflect a metabolic shift in FRDA

A downregulation of proteins specific of type 1 or slow-twitching fibers was observed in FRDA, including contractile proteins such as the myosin heavy chains MYH7 and MYH7B (Schiaffino, 2018), the troponin TNNT1 (Wei et al., 2014), and the calcium transporter ATP2A2 (Brandls et al., 1987). Lower expression of the enzyme nicotinamide nucleotide transhydrogenase NNT in FRDA probes is a further hallmark of type 2 fast-twitching fibers as compared to type 1 fibers (Schiaffino et al., 2015a). In the previously performed histology, predominance of type 2 fibers was evident in 2/5 patients, while the most evident findings in the other 3/5 was grouping of type 1 and 2 fibers. In all five patients, atrophic changes involved both type 1 and 2 fibers. Furthermore, we found a downregulation of the embryonic isoform MYH3 (Schiaffino, 2018) as compared to controls. Developmental myosins are re-expressed during muscle regeneration, providing a marker of regenerating fibers in an affection of skeletal muscle (Schiaffino et al., 2015b). Also MYH6, a cardiac myosin (Schiaffino, 2018), listed among the downregulated DE proteins in FRDA. Two enzymes of the mitochondrial beta-oxidation pathway, CPT1B and ACAT1 were also downregulated in FRDA. CPT1B is a muscle specific isoform of carnitine palmitoyltransferase, the rate-controlling enzyme of the long-chain fatty acid beta-oxidation (Van Der Leij et al., 2002). Mitochondrial fatty acid oxidation is the main muscle energy source during endurance exercise (Hénique et al., 2015). Loss of muscle carnitine palmitoyltransferase in mice induces a shift from slow-twitch to fast-twitch glycolytic fibers (Pereyra et al., 2022), while enhancement of mitochondrial fatty acid oxidation via CPT stimulation induces a shift toward oxidative fibers (Hénique et al., 2015). ACAT1 catalyzes the last step of the beta-oxidation pathway, which produces acetyl-CoA and a fatty acyl-CoA (Qian et al., 2020).

### Upregulation of antioxidative defense enzymes

The only upregulated protein in FRDA was the glutathione S-transferase mu 4 (GSTM4), an enzyme involved in detoxification from electrophilic compounds and oxidative stress (Comstock et al., 1993). Considering the global shift toward downregulation and the restriction of the ribosomal proteins pool in FRDA, we speculated that eventual upregulation events might display a smaller size effect. Applying less stringent criteria (*p* < 0.1, instead of *p* < 0.01, fold change >1.5), a list of 104 proteins more abundant in FRDA as compared to controls was generated. STRING based interactome analysis evidenced two clusters: a group of antioxidative defense enzymes (beyond GSTM4 also GSTM3, GPX7, ADHC1, and SOD3) on the other hand proteins related to intracellular signaling (MAPK1, CASP3, CALM3, YWHAQ, and YWHAB) (see Supplementary Figure 1).

### Comparison with transcriptomics data

Previously published RNA-sequencing data from the same samples (Indelicato et al., 2023) showed widespread differences in global transcriptome in FRDA as compared to controls. As expected, differences at proteomics level are less pronounced but generally consistent with the pathways highlighted by RNA-sequencing. FRDA skeletal muscle proteome retained the widespread downregulation in OXPHOS elements which was detected at RNA-sequencing. Several genes related to ISC biosynthesis were downregulated at mRNA level, while the corresponding proteins were not DE in FRDA as compared to controls. Leptin mRNA was strongly upregulated in FRDA in our previous study (Indelicato et al., 2023). Though, we did not detect leptin via MS likely due to his function as secreted paracrine factor. Interestingly, mRNAs of a few downregulated DE proteins were in contrast upregulated in FRDA transcriptomes (PHB2, MT-ND6, TMEM11, and MYH3). These findings underscore a complex level of regulation with modulation and compensatory events both at a translational and transcriptional level (Liu et al., 2016). In line with the transcriptome profiles, a large cluster of downregulated ribosomal proteins emerged in the present analysis (Figure 3), reflecting a global shift toward repression of protein synthesis in the skeletal muscle in FRDA.

## Discussion

Skeletal muscle proteome analysis from affected FRDA patients confirms the key mitochondrial signature of the disease and unveils its multifaceted aspects. Indeed, expression changes in FRDA proteomes encompass not only an extensive downregulation of OXPHOS proteins, but also alterations in regulatory/signaling pathways governing cytoarchitectural organization and mitochondrial fission/fusion. Impairment of OXPHOS was first delineated in a milestone work demonstrating reduced activity of ISC containing mitochondrial enzymes in FRDA cardiac muscle (Rotig et al., 1997). Cumulative evidence from animal models and *in vivo* studies corroborated a failure of the OXPHOS in FRDA (González-Cabo and Palau, 2013). Beyond this established event, an earlier case report (Gallagher et al., 2002) as well as recent studies in murine models (Vásquez-Trincado et al., 2022) and organoids (Mazzara et al., 2020) revealed alteration of the mitochondrial ultrastructure (Vásquez-Trincado et al., 2022). In line with these findings, we showed in FRDA a downregulation of key components of the MICOS complex, which regulates mitochondrial morphology. Moreover, several subunits of complex V in FRDA skeletal muscle were downregulated. Complex V assembles in modular oligomeric structures at the mitochondrial inner membrane, thus contributing to the shaping of cristae (Paumard et al., 2002). Monogenic complex V defects resulting in reduced protein level are invariably accompanied by altered mitochondrial ultrastructure (Oláhová et al., 2018). Moreover, several DE proteins involved in mitochondrial fusion/fission and mitophagy emerged in our samples, a finding consistent with an impairment of mitochondrial regeneration mechanisms.

The present analysis further evidenced a few changes which are characteristics of a slow- to fast-type fibers conversion in FRDA skeletal muscle (Schiaffino, 2018). Slow-twitch or type 1 fibers sustain prolonged exercise during endurance. They are rich in mitochondria and rely mostly on fatty acids oxidation for energy supply (Schiaffino, 2018). Fast-type or type 2 fibers are glycolytic muscle fibers which have a lower mitochondrial content and are less dependent on oxidative pathways. Shift to type 2 fibers predominancy is a common finding in mitochondrial myopathy (Lu et al., 2019) which may explain reduced exercise tolerance (Wei et al., 2014). Similar changes were detected at a transcriptomic level in a mouse model of FRDA (Coppola et al., 2009), while in our patient-derived skeletal muscle samples they were detected only at a protein level (Indelicato et al., 2023).

Up to date, few proteomics studies have been conducted in FRDA patients derived tissues, namely in lymphocytes (Télot et al., 2018), fibroblasts (Napierala et al., 2021), cerebrospinal fluid (Imbault et al., 2022), and in iPSC-derived proprioceptors enriched neuronal cultures (Dionisi et al., 2023). Clinically non-affected cells, such as FRDA patients-derived lymphocytes and fibroblasts, displayed milder changes in mitochondrial proteome and, interestingly, a much stronger downregulation of frataxin (Télot et al., 2018; Napierala et al., 2021). Frataxin downregulation was less pronounced in iPSC-derived proprioceptors (Dionisi et al., 2023), similarly to skeletal muscle. These findings underscore the peculiar susceptibility of specific tissues to a relatively mild deficiency of frataxin, an unusual circumstance for an autosomal recessive disorder (Lynch et al., 2012). Earlier studies applying different techniques revealed a much more marked decrease of frataxin protein level in the heart and in nucleus dentatus in FA necropsies (Koeppen et al., 2015a,b). Beyond methodological issues and tissue-related differences, the discrepancy between ours and previous findings may be explained by (1) a less severe genotype in our patients and (2) a progression of frataxin deficit during life. Indeed, pathologically expanded GAA allele are subjected to somatic instability in affected tissues. This would predict a more marked frataxin downregulation in necropsies as compared to *in vita* collected samples or iPSC-derived cells. Our finding underscores that a mild *FXN* upregulation via gene therapy or genome editing might be sufficient to restore frataxin deficit in the skeletal muscle. Susceptibility to frataxin deficiency likely depends also on the time window in which it occurs (Koeppen et al., 2017). Indeed a strong developmental component has been highlighted in the pathophysiology of FRDA (Eigentler et al., 2013; Koeppen et al., 2017). In spite of this, FRDA symptoms manifest several years after birth and do steadily progresses over time, implying the involvement of additional pathogenic mechanisms besides frataxin deficiency. Delineating the factors sustaining progression is an unmet goal which is relevant to drug development (Indelicato and Bösch, 2018). Indeed, these superimposing stressors might be more responsive to pharmacological intervention than developmental issues. Therapeutic strategies acting downstream in the pathophysiological cascade may be effective also in patients in advanced stages, who would not benefit from future gene therapy (Indelicato and Bösch, 2018). Especially a cumulative mitochondrial damage has been advocated as possible progression driver (Singh et al., 2015; Karthikeyan et al., 2023). Skeletal muscle from FRDA patients with longstanding disease display extensive changes in mitochondrial transcriptome and proteome. Conversely, mitochondrial phenotype (Lai et al., 2019; Dionisi et al., 2023), as well as the global degree of protein downregulation (Dionisi et al., 2023), is far less pronounced in iPSC derived neurons, likely reflecting the biological state of an early disease stage. These considerations emphasize the value of skeletal muscle -omics as a window into the evolving pathophysiology of FRDA. Notably, a majority of DE proteins identified by the present study are target of the transcription factor NRF2, the master regulator of cellular redox homeostasis. Very recently, the compound omaveloxolone, a NRF2 inducer, was approved by FDA as first drug for the treatment of FRDA (Lee, 2023). Omaveloxolone treatment resulted in a mild, but consistent, improvement in the neurological scale mFARS as compared to placebo (Lynch et al., 2021). The milestone set by omaveloxolone approval encourages to pursue therapeutic strategies targeting mitochondria.

Our present findings add to the scarce, but consistent, literature which corroborate a skeletal muscle phenotype in FRDA (Lodi et al., 1999; Bradley et al., 2000; Vorgerd et al., 2000; Coppola et al., 2009; Sival et al., 2011; Nachbauer et al., 2012; Vásquez-Trincado et al., 2022). Myopathy may relevantly contribute to disability in advanced stages. This issue, along with the accessibility of skeletal muscle compared to the nervous tissue, should prompt the evaluation of *ad hoc* pharmacological interventions which may enrich the future palette of FRDA therapeutics (Lynch and Farmer, 2021).

Our study bears severe limitation, first of all the small sample size. Despite the feasibility, skeletal muscle biopsy is still an invasive procedure comparing to blood sampling and thus pilot studies are justified before future application in larger cohorts. Due to the small sample, which included only adults, the present results may not be generalizable to other disease subgroups, especially children. For the same reason, the study was not powered to detect correlation of DE changes with the *FXN* levels. We could not further infer on the previously detected leptin mRNA upregulation in FRDA skeletal muscle since the current protocol was designed to detect intracellular proteins. We furthermore did not address the possibility of heterogeneous findings in different fiber subsets.

Findings from proteomics do not binarily match RNA-sequencing data (Liu et al., 2016), but are the closest to the final biological picture which is supposed to drive the clinical phenotype. Our data confirm a predominant mitochondrial biosignature of FRDA, with extensive involvement of the OXPHOS, but also bring into focus a derangement of mitochondrial architecture and maintenance pathways. The present findings are relevant for the design of future therapeutic strategies and highlight the value of skeletal muscle-omics as disease state readout in the setting of a mitochondrial, neuromuscular disorder such as FRDA.

## Data availability statement

The datasets presented in this study can be found in online repositories. The names of the repository/repositories and accession number(s) can be found below: the MS-proteomics data have been deposited to the ProteomeXchange Consortium (http://proteomecentral.proteomexchange.org) via the PRIDE partner repository with the dataset identifier PXD044554.

## Ethics statement

The studies involving humans were approved by the Ethikkommission der Medizinischen Universität Innsbruck and the local Ethic Committee (Approval number, UN 3152_LEK). The studies were conducted in accordance with the local legislation and institutional requirements. The participants provided their written informed consent to participate in this study.

## Author contributions

EI: Conceptualization, Data curation, Visualization, Writing – original draft, Writing – review and editing. KF: Data curation, Formal analysis, Methodology, Visualization, Writing – review and editing. MA: Project administration, Writing – review and editing. WN: Resources, Writing – review and editing. RS: Conceptualization, Supervision, Writing – review and editing. JW: Data curation, Supervision, Writing – review and editing. BS: Data curation, Investigation, Methodology, Validation, Writing – review and editing. SB: Funding acquisition, Resources, Supervision, Writing – review and editing.

## Footnotes

1
http://proteomecentral.proteomexchange.org
