# Supplementary material for: Skeletal muscle proteome analysis underpins multifaceted mitochondrial dysfunction in Friedreich’s ataxia
Source: Front Neurosci. 2023 Oct 31;17:1289027. doi: 10.3389/fnins.2023.1289027 (PMC10644315; doi:10.3389/fnins.2023.1289027)

Supplementary Figure 1: Interactome of the upregulated proteins in FRDA patients (at $p<0.1$) adapted from the STRING database.

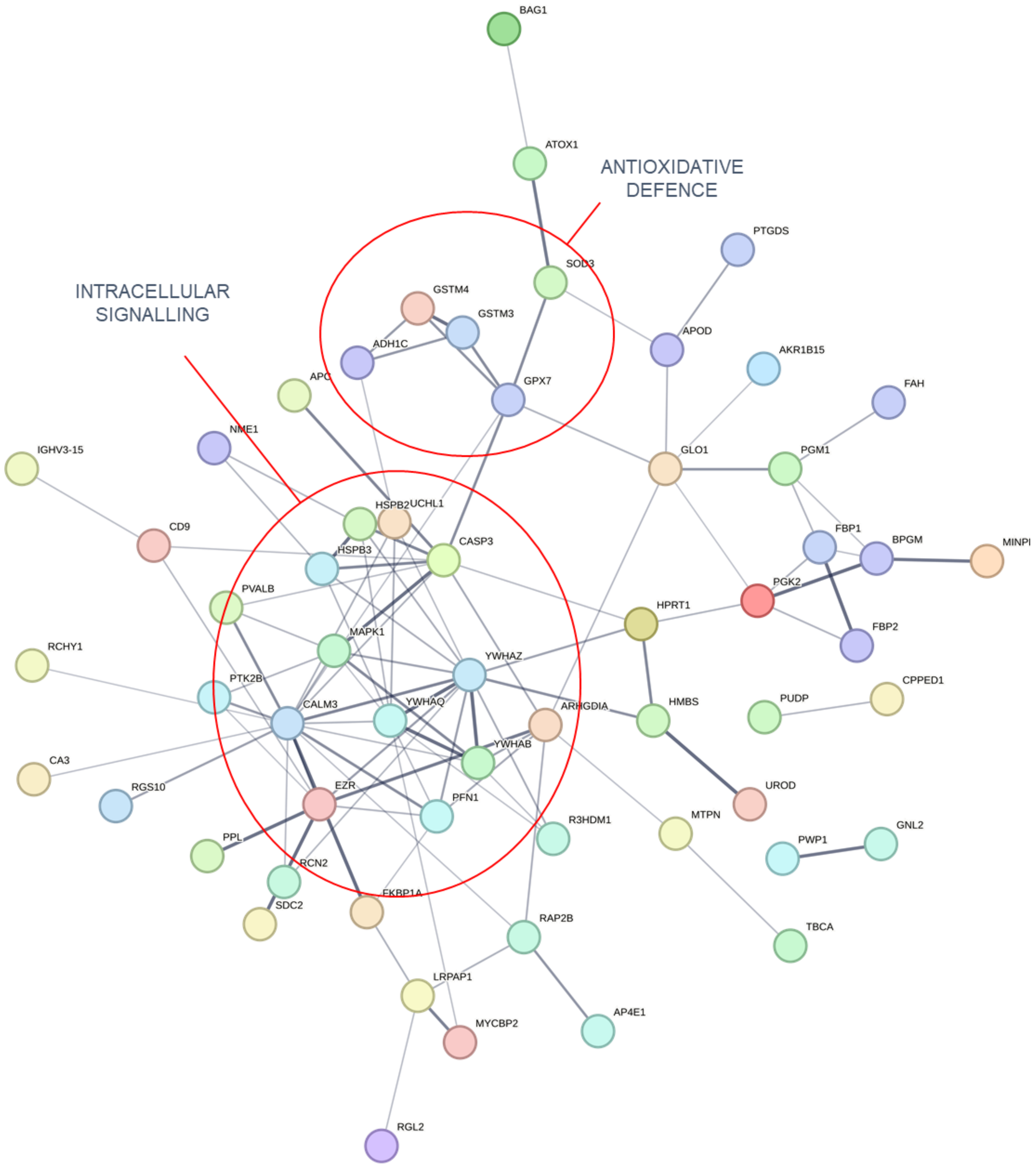

Supplement: Supplementary file 2 [file Image_1.pdf]
